# Pain Management Research in Prehospital Settings: The Pain of Exception from Informed Consent

**DOI:** 10.1002/eahr.70007

**Published:** 2025-12-31

**Authors:** Michael Linke, Francis X. Guyette, Jason McMullan

**Affiliations:** ^1^ Adjunct professor in the Department of Internal Medicine at the University of Cincinnati; ^2^ Professor in the Department of Emergency Medicine at the University of Pittsburgh; ^3^ Professor in the Department of Emergency Medicine at the University of Cincinnati

**Keywords:** human subjects research, human research ethics, human research regulations, emergency medicine research, pain management, ketamine, fentanyl, trauma, injury

## Abstract

Effective pain management in prehospital settings remains a significant challenge, particularly for patients with acute trauma. This report examines the complexities of conducting prehospital pain studies, focusing on two clinical trials as case studies. The first trial implemented a novel two‐step informed consent process, which, despite its innovation, faced logistical challenges and introduced biases favoring the enrollment of less severely injured patients. The second study, conducted under the federal regulatory pathway for Exception from Informed Consent (EFIC), successfully addressed consent challenges but restricted enrollment to severely injured patients to meet EFIC regulatory requirements. Both studies excluded women of childbearing age due to concerns about fetal safety. These limitations underscore the urgent need for regulatory revisions to expand research with alteration of informed consent, enabling the development of new interventions for prehospital pain management.

Effective treatment of acute pain after traumatic injuries remains a significant clinical challenge, particularly in the prehospital setting. Paramedics must rapidly assess the patient, perform life‐saving interventions (e.g., tourniquet placement), choose the most appropriate hospital destination, and prepare the patient for transport within 10‐15 minutes of arriving at the scene of injury. Analgesia is a critical component of injury care, providing relief from suffering and facilitating painful lifesaving procedures and extrication from the scene. Compared to the multiple nurses, technicians, and physicians caring for a patient in the trauma bay in most emergency departments, only one or two paramedics are rendering aid to the same patient in the back of an ambulance. Given the significant time and resource constraints of the prehospital setting, the opportunity cost of every action must be considered by the paramedics, and in‐hospital practices do not easily translate into the field. Emergency department‐based clinical trials generally exclude participants who received prehospital analgesia, introducing a wellness bias and limiting generalizability.^1^, ^2^, ^3^, ^4^, ^5^ In order to provide the best care for trauma patients prior to hospital arrival, high‐quality clinical trials performed by prehospital clinicians in the prehospital environment are essential.

A major obstacle to conducting research on acute pain due to traumatic injuries in the prehospital environment is the difficulty in obtaining informed consent from patients in the emergency setting. While informed consent can sometimes be obtained from patients with acute pain, those in the most severe pain may not be able to fully consider the risks, benefits, and alternatives of a clinical trial. Furthermore, delaying interventions for pain to obtain consent may be seen as unethical or coercive and the time taken by paramedics to obtain informed consent for research may delay or distract from other clinical activities. Administration of narcotic analgesics, the current clinical standard, may impair a patient's ability to provide informed consent.^6^

Based on the setting and acuity of the patient's condition, prospective interventional prehospital pain trials are considered more than minimal risk and are not eligible for alterations to or waivers of informed consent.^7^ Many pain medications routinely used by paramedics have not been approved by the US Food and Drug Administration (FDA) for the condition being treated (e.g., ketamine) or the route of administration (e.g., intranasal), and any study conducted under an FDA Investigational New Drug (IND) protocol is, by definition, associated with more than minimal risk.^8^ This is usually true even for comparative effectiveness studies of interventions that are considered standard care. Clinical trials in the emergency setting that pose more than minimal risk and for which patients do not have capacity to provide informed consent to participate may be conducted under an Exception from Informed Consent (EFIC) when certain conditions are met.^9^ EFIC applies to studies of time‐dependent treatments of life‐threatening conditions where existing therapies are unsatisfactory, standard informed consent cannot be obtained, potential participants cannot be identified beforehand, and the intervention must demonstrate the potential for direct benefit to the patient‐participant. EFIC requires the investigators to conduct community consultation and public disclosure prior to trial initiation to demonstrate that the trial is morally, ethically, and socially acceptable in the community. Public disclosure continues throughout the trial for transparency and concludes with sharing of study results to the community. Even though EFIC allows patients to be enrolled in a study without their prior informed consent, after they are enrolled in the study, the patients are approached as soon as is feasible to inform them that they being treated in a research study and to obtain their informed consent for continued participation in the study. Prehospital interventions for out‐of‐hospital cardiac arrest,^10^ status epilepticus,^11^ traumatic brain injury,^12^ hemorrhagic shock,^13^ and stroke^14^ were evaluated under EFIC regulations. Clinical trials on acute pain in the prehospital setting do not meet this threshold, as pain is not considered life‐threatening, and current care is considered satisfactory for most patients.

On March 12, 2024, the National Institutes of Health (NIH) convened a workshop in Bethesda, Maryland, to explore the challenges of emergency care research and informed consent. The goal of the workshop was to bring together emergency care research stakeholders from diverse backgrounds and perspectives to share their experiences and opinions on current and future EFIC policies and procedures. Two clinical trials targeting the treatment of traumatic pain were offered as case studies to explore the nuances and challenges of conducting research in the prehospital setting. The

**The authors advocate for further amending the current EFIC regulations or alteration of informed consent requirements to permit the approval of clinical studies involving conditions that may not be life‐threatening, but where obtaining informed consent from patients or their surrogates is impractical.**

goal of this paper is to describe the ethical limitations of prehospital pain research through these case studies and to identify opportunities for improving prehospital research processes.

## CASE STUDIES

The principal investigators (FG, JM) of two contemporary prehospital pain studies were invited to present their experiences as part of the workshop, and the chair (ML) of an institutional review board (IRB) responsible for oversight of one of the trials provided regulatory and ethical perspectives. These two pain trials served as case studies to highlight the challenges of obtaining informed consent to participate in research in the prehospital setting, as well as the complexities of implementing EFIC.

The first study, Intranasal Ketamine as an Adjunct to Fentanyl for the Prehospital Treatment of Acute Traumatic Pain (Intranasal Ketamine Study),^15^ investigated the efficacy of adding intranasal ketamine to fentanyl for prehospital analgesia in trauma patients.^16^ In this study, paramedics screened patients for study enrollment, obtained informed consent to participate, and administered the investigational medications to participants in the field. Since the IRB and the FDA determined that the study involved more than minimal risk, it did not meet the criteria for waiver or alteration of informed consent under federal regulations. Additionally, the EFIC pathway was not an option for the study, as the eligible patient population would not be considered in a life‐threatening situation that necessitates urgent intervention. To provide a practicable informed consent process that could be used in the prehospital setting and allow follow‐up of the participants, the investigation was conducted as two separate studies, a “prehospital study” and a “follow‐up study.” Each study utilized a separate consent form. In the “prehospital study,” paramedics used a streamlined three‐page consent form that incorporated all required consent elements to obtain informed consent from patients in the field for randomization, administration of the study drug, and initial outcome assessments. After hospital admission and stabilization, emergency department‐based clinical research coordinators approached participants to provide informed consent for secondary and distal outcomes by using a second, separate consent form, which included Health Insurance Portability and Accountability Act authorization language, for the “follow‐up study.”^17^

The study was successfully completed using this informed consent process; however, several challenges arose during the study's implementation in the prehospital setting, leading to some instances of protocol noncompliance. Clinical research and human subjects protection training are not a part of initial or continuing paramedic education, and clinical care in the prehospital setting is usually provided under the premise of “implied consent” and clinical protocols developed by a physician medical director. Several hundred paramedics underwent targeted education on the concept of informed consent for research, the research protocol (which differed from clinical protocols), and the signature requirements for the informed consent document (which differed from those for patient signatures on clinical documentation). The most common protocol deviations occurred when study medication was administered to patients who did not meet the research protocol's inclusion/exclusion criteria but could be treated under the clinical protocols. The most common compliance issue with consent documentation was failing to have the participant independently date the informed consent form after signing. Unique cases included paramedics allowing a paralyzed man's legal power of attorney to sign the consent form and accepting verbal consent by a man incapable of holding a pen due to significant injuries to both arms and hands. In all cases, the informed consent process was followed, even if documentation of consent did not meet current standards. However, corrective action plans required additional ongoing training to minimize bias in allocation and improve consent documentation for all involved paramedics, at a significant cost and labor. The efforts needed to support compliant prehospital informed consent are not sustainable and unlikely to be reproducible at scale.

Additionally, the logistical difficulties of obtaining informed consent in emergency conditions appeared to bias the results toward less severely injured patients. A significant number of patients were deemed ineligible by paramedics to participate in the study, either due to critical illness or a perceived inability to provide informed consent, further impacting study enrollment and generalizability.^18^ Another significant challenge in conducting research in the prehospital setting is safely enrolling women of childbearing potential in studies that may pose a risk to the fetus, as there is no qualitative pregnancy test appropriate for prehospital use. In this study, the FDA expressed concerns about potential harm to a fetus from ketamine exposure and did not accept self‐report of possible pregnancy as an acceptable screening strategy. To mitigate this risk, women of childbearing age were excluded from the study due to the impracticality of conducting pregnancy testing in the field. Consequently, the investigators decided to exclude all women to avoid potential bias that could result from including only women over 50, particularly given the study's upper age limit of 65.

The second study, the Prehospital Analgesia INtervention Trial (PAIN),^19^ compares the administration of intravenous fentanyl to intravenous ketamine in patients with suspected hemorrhagic shock in the prehospital setting. The PAIN study team recognized the challenges of enrollment with informed consent in the Intranasal Ketamine Study and sought approval to conduct the study under EFIC. To meet the EFIC criteria, the inclusion and exclusion requirements were adjusted, restricting enrollment to severely injured patients with evidence of compensated shock (Shock Index >0.9). This population was selected to align with the EFIC requirements for life‐threatening injury (severe trauma), to emphasize the unsatisfactory nature of the standard therapy (fentanyl), and the potential direct benefit of the intervention (ketamine). In patients with compensated shock, fentanyl may contribute to hypotension, hypoxia, or the need for advanced airway management. Alternatively, ketamine may be less likely to cause hypotension and does not suppress the respiratory drive. To comply with EFIC regulations and ensure that the risks of the investigation were reasonable in relation to the potential benefits of the study intervention, patients were excluded from participation if they had no evidence of shock, as standard care (fentanyl) would be satisfactory. To follow local protocols regarding analgesic administration, patients with frank hypotension or altered mental status were also excluded from the study, as any analgesic could pose an unacceptable risk to the patient. As with the Intranasal Ketamine Study, during their review of the protocol, the FDA raised concerns about potential fetal exposure to ketamine. Initial and subsequent efforts to define screening of women of childbearing age to include those at the lowest risk of pregnancy were also rejected. To address this issue, the study team excluded female patients under the age of 50. The investigators suggested both screening for self‐reported pregnancy and utilizing a screen (derived from the Centers for Disease Control and Prevention's Practice Recommendations) with a negative predictive value >99% for pregnancy.^20^ Both strategies were deemed insufficient to exclude pregnancy. Ultimately, the FDA determined that the protocol, modified to restrict the population to patients with compensated shock and exclude women of childbearing age, met the criteria for EFIC.

## DISCUSSION

Each of the clinical trials discussed found individual solutions to the challenges of performing prehospital studies of pain management after trauma. Those solutions may reduce the generalizability of the study findings. We presented these concerns at the NIH workshop, where attendees discussed the broader implications for subsequent research and highlighted remaining ethical and regulatory dilemmas.

### Use of EFIC for pain studies

Relief from suffering is a cornerstone of compassionate care, no matter what the setting, and a clinician uses best judgment in treating patients. Research and regulations guiding informed consent force subjective categorization of the same patients: those without life‐threatening injuries who are not overly distracted by pain and can provide informed consent, those without life‐threatening injuries who cannot provide informed consent, and those with life‐threatening injuries. This leaves researchers with a dilemma: either obtain informed consent from the least severely injured patients, as seen in the Intranasal Ketamine Study, or conduct EFIC‐approved studies, such as PAIN, on the most severely injured patients, thereby missing the middle group of trauma patients experiencing moderate to severe pain who are not in shock and thus do not meet EFIC eligibility criteria for enrollment in a study. Since this group of patients is not critically unstable and is a common prehospital population, they may also be the most likely to benefit from new treatments for acute pain and produce findings that are most generalizable to the broader population of prehospital patients. Even though these patients are not critically unstable, they would often still be unable to provide informed consent to participate in a study using a consent form that meets all regulatory criteria and could not be enrolled in studies that do not qualify for EFIC.

EFIC approval for the PAIN study is certainly a positive development, because it allows evaluation of treatment in patients with a life‐threatening condition, instead of previous EFIC trials that studied interventions for life‐threatening conditions. The authors advocate for further amending the current EFIC regulations or alteration of informed consent requirements to permit the approval of clinical studies involving conditions that may not be life‐threatening, but where obtaining informed consent from patients or their surrogates is impractical, under EFIC provisions utilizing community consultation, public disclosure, and post‐intervention informed consent for continued participation in the trial. This change, possibly in conjunction with a broader spectrum of informed consent processes utilized in the RECOVER IV trial of mechanical cardiac support in patients with cardiogenic shock would ensure that studies maintain ethical standards.^21^ To accommodate the RECOVER IV trial's 30‐minute therapeutic window, 15 minutes was designated for obtaining prospective informed consent from the patient or their legally authorized representative (LAR) before enrolling them in the study. Since it was unlikely that informed consent could be obtained within this limited timeframe, even using a streamlined consent form and process such as that used in the Intranasal Ketamine Study, patients or their LARs were also provided with an opportunity to decline participation after a brief explanation using a study‐specific script which contained similar information as that typically seen in the “Key Information” section required in the consent forms by the revised Common Rule.^22^ Allowing an “opt‐out” preserved the patient's autonomy, even in situations where obtaining informed consent was not possible.

### Exclusion of potentially pregnant participants

The two case studies highlight significant concerns regarding the exclusion of female participants due to potential pregnancy, emphasizing the ethical and clinical need to balance protecting pregnant individuals and their fetuses as a vulnerable population with providing optimal, evidence‐based care tailored to their needs. Ethically, excluding women under age 50 prioritizes the protection of a potential fetus over understanding how the treatment affects nonpregnant women in this age group. Clinically, it was noted that ketamine is frequently used in pregnant patients in various medical settings, including prehospital pain management, creating a disincentive to performing the clinical trials necessary to demonstrate its safety, as there is no need for a manufacturer to obtain a specific indication from the FDA. Excluding potentially pregnant participants limits the opportunity to gather valuable data and insights from women in general in this population.

Excluding women of childbearing age from these studies does not prevent them from receiving these pain medications in practice; it merely ensures that clinicians will administer these therapies without a clear understanding of the associated risks. Regulators and investigators should actively strive to include all women in studies and avoid their exclusion unless supported by valid scientific justification.^23^ Alternatively, the development of ultra‐rapid point‐of‐care pregnancy testing platforms using capillary “fingerstick” blood samples, similar to glucometers or electrochemical tests that utilize whole blood samples, could provide a reliable and efficient alternative. These advancements would help eliminate the categorical exclusion of females of childbearing age, ensuring broader inclusion in research and clinical decision‐making while maintaining rapid and accurate pregnancy detection. It is important to emphasize that standard care is essentially an uncontrolled experiment, offering little to no valuable insights.^24^

Categorically excluding females of childbearing age who could otherwise provide informed consent does not uphold the principle of autonomy. Informed consent requires participants to weigh the risks and benefits of enrolling in a clinical trial, considering any potential implications for a fetus through a personal lens. Some may prioritize avoiding risk to a potential fetus in a case of possible pregnancy and choose not to participate, while others may prioritize potential individual benefit and enroll in a trial. Objectively testing for pregnancy provides additional information to be considered during the informed consent process, but completely ignoring self‐report (as initially proposed to the FDA by the Intranasal Ketamine Study investigators) does little to instill trust between participants and researchers. Clinicians frequently rely on self‐report of pregnancy during the initial phases of care, and researchers rely on self‐report of medication allergy and other inclusion/exclusion criteria before trial enrollment.

### Paramedic participation in prehospital clinical trials

In the Intranasal Ketamine Study, over 300 paramedics were considered key personnel, significantly increasing the IRB's oversight burden. Because paramedics were responsible for conducting the informed consent process and administering the study intervention, they were required to complete formal human subjects protection training as well as study‐specific protocol training. The IRB at the University of Cincinnati approved tailored training materials for the paramedics and reviewed a delegation log during continuing review to confirm that all paramedics had completed the required training. Special processes were developed to maintain records outside of the university's electronic reporting system, as none of the paramedics had profiles because they were not otherwise affiliated with the university.

In contrast to the Intranasal Ketamine Study, for the PAIN study it was determined that the principal investigators and the local EMS agency medical directors were key personnel who were required to complete formal human subjects training. The paramedics were solely providing care and following a research protocol; therefore, they were not classified as key personnel. As a result, the University of Pittsburgh IRB did not face an increased oversight burden. Participating paramedics underwent abbreviated training on human subjects research, which consisted of a narrated presentation and a mandatory quiz. Training time for paramedics is limited, and the PAIN approach allowed for a more focused study of procedures and interventions. Notably, the Community Research Action Board at the University of Pittsburgh supported paramedic participation in the PAIN study, particularly because it ensured the inclusion of participants from urban areas, not just suburban communities.

In the Intranasal Ketamine Study, participating paramedics noted several barriers and facilitators to conducting prehospital research. Paramedics generally valued research and supported conducting trials in the prehospital setting. Most concerns involved the responsibility of obtaining informed consent. Initiating conversations about research, not having adequate time, and obtaining an ink signature were commonly cited challenges, and half preferred to use an enrollment script. The use of a standardized video to assist the informed consent process and the use of electronically recorded signatures were suggested as future improvements.^25^ While these are attractive concepts, and many have been supported by ethicists and/or used in non‐US trials,^26^, ^27^ none are currently allowed by the relevant regulations governing research with humans.^28^, ^29^ The use of a script is allowed only for those unable to read and requires a co‐signature by an uninvolved third party; the FDA also requires handwritten signatures on paper.

An additional unique challenge of the PAIN study was the restriction imposed by Pennsylvania law, which permits only physicians to obtain informed consent from patients for participation in drug studies. This legal limitation would have made it impossible for paramedics to obtain informed consent in the field in Pennsylvania, preventing the study from being conducted if informed consent had been required.

## SUMMARY

This report highlights the challenges of conducting pain research in prehospital settings, particularly regarding the informed consent process. It is encouraging that insights gained from the Intranasal Ketamine Study were used to justify the application of EFIC in the PAIN study, streamlining the research process in an already complex environment. In the absence of revisions to the current EFIC regulations, investigators can utilize the existing regulatory flexibility^30^ to demonstrate that their prehospital pain studies comply with all ethical standards and regulatory requirements for research under EFIC, as was done with the PAIN study. Working with the FDA can help ensure these studies are appropriately designed and ethically justified within the current EFIC framework.
